# Nordic treatment guidelines for rare epileptic conditions: A literature review

**DOI:** 10.1002/brb3.2622

**Published:** 2022-06-28

**Authors:** Kishan Vyas, Hannah Luedke, Benjamin Ruban‐Fell

**Affiliations:** ^1^ GW Pharmaceuticals (A Jazz Pharmaceuticals company) London UK; ^2^ Costello Medical Cambridge UK

**Keywords:** CDKL5 deficiency disorder, Lennox‐Gastaut syndrome, myoclonic epilepsies, practice guidelines, Rett syndrome, tuberous sclerosis complex

## Abstract

**Introduction:**

The onset of severe, drug‐resistant seizures in early childhood is characteristic of the rare epileptic disorders Lennox‐Gastaut syndrome (LGS), Dravet syndrome (DS), and CDKL5 deficiency disorder (CDD) and is frequently observed in the rare genetic conditions tuberous sclerosis complex (TSC) and Rett syndrome (RTT). High‐quality treatment guidelines are needed for optimal management of these conditions. This review aimed to assess content, availability, and development of treatment guidelines for these disorders in the Nordics region (Denmark, Finland, Iceland, Norway, and Sweden).

**Methods:**

A targeted literature review (TLR) was therefore conducted in November/December 2020 by manually searching online rare disease and guideline databases in addition to relevant health technology assessment and regulatory agency websites to identify pharmacological treatment guidelines for DS, LGS, TSC, RTT, and CDD. Search terms for each disorder were translated to identify country‐specific guidelines. Treatment recommendations, geographical focus, and guideline development methodology was extracted into a predetermined extraction grid.

**Results:**

Most of the 24 eligible guidelines identified (16/24; 66%) were specific to particular countries; Sweden was the most represented (7/24 [29%] guidelines), while no guidelines were identified for Iceland. Guideline development methodologies were heterogeneous, including systematic literature reviews/TLRs and expert consultation; several methodologies did not report details on the evidence sources used (7/24 [29%] guidelines). Treatment recommendation availability was variable across disorders, ranging from 126 treatment recommendations (LGS) to none (RTT, CDD).

**Conclusion:**

Comprehensive, consensus‐based treatment guidance developed via international collaboration within the Nordics region is necessary to optimize patient care in these five rare epileptic conditions.

## Graphical abstract

The onset of severe, drug‐resistant seizures in early childhood is characteristic of the rare epileptic disorders Lennox‐Gastaut syndrome, Dravet syndrome, and CDKL5 deficiency disorder and is frequently observed in the rare genetic conditions tuberous sclerosis complex and Rett syndrome. International collaboration to develop high‐quality treatment guidelines is needed for optimal management of these conditions. This review aimed to assess content, availability, and development of treatment guidelines for these disorders in the Nordics region (Denmark, Finland, Iceland, Norway, and Sweden).

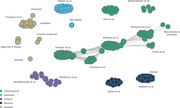

## 1 INTRODUCTION

Dravet syndrome (DS), Lennox‐Gastaut syndrome (LGS), and CDKL5 deficiency disorder (CDD) are rare, severe, treatment‐resistant developmental and epileptic encephalopathies with distinct etiologies (Camfield, 2011; Gataullina & Dulac, 2017; Olson et al., 2019). These conditions are characterized by seizure onset in early childhood, which may lead to progressive cerebral dysfunction as well as severe behavioral and cognitive impairments (Jain et al., 2013; National Organization for Rare Disorders, 2020a). Although CDD predominantly affects females, many male patients have been identified and may have more severe symptoms (National Organization for Rare Disorders, 2020a). Severe seizures in children are also common in the rare genetic disorders Rett syndrome (RTT) and tuberous sclerosis complex (TSC) (Nabbout et al., 2019; Nissenkorn et al., 2015; Curatolo et al., 2018; Nissenkorn et al., 2010). RTT is a progressive neurodevelopmental disorder presenting with autism, epilepsy, and intellectual disability that mainly affects females (Khwaja & Sahin, 2011; National Organization for Rare Disorders, 2015), while TSC is a multisystem disorder caused by benign tumor formation, leading to complications including cognitive dysfunction and developmental delay (Khwaja & Sahin, 2011; National Organization for Rare Disorders, 2019).

Effective seizure management is required in epileptic conditions to prevent injury, disability, and life‐threating complications including sudden unexpected death in epilepsy (SUDEP) (Krauss & Sperling, 2011). Many pharmacological treatments targeting seizures are associated with significant side effects, meaning that epileptic seizure management requires careful therapy selection to improve patient quality of life (QoL) (Krauss & Sperling, 2011; Mitchell et al., 2012). While nonpharmacological interventions such as dietary modification (typically ketogenic diet), and in some instances, surgery (e.g., vagal nerve stimulation) aim to reduce seizure frequency and severity (Krauss & Sperling, 2011; Mitchell et al., 2012; Pavan et al., 2017), symptomatic and supportive medical management with long‐term antiseizure medications (ASMs) remains the mainstay of epilepsy treatment (Brown, 2016).

However, seizure management in these disorders is challenging even with widespread ASM use. Seizures in all five conditions are often treatment resistant (i.e., are not adequately controlled despite use of two or more appropriate ASMs), with patients often failing to achieve complete seizure control (Kaur & Christodoulou, 2019; Krauss & Sperling, 2011; Mitchell et al., 2012; National Organization for Rare Disorders, 2018, 2020a, 2020b; Northrup et al., 2018; Pavan et al., 2017). Furthermore, individual response to medication can be variable, and ASMs may become less effective with time or worsen seizure control in some instances (Brown, 2016). Moreover, identifying and selecting appropriate ASMs can be difficult, especially with ongoing research and drug approvals constantly changing the landscape of available treatments. Clinical practice guidelines can help guide treatment decisions and ensure that clinicians have an evidence‐based approach to seizure management (Guyatt et al., 2008). Treatment guidelines can also be used to inform other key documents that play a role in patient access to novel treatments, such as health technology assessments (HTAs) and guidance by regulatory bodies (Akehurst et al., 2017; Detela & Lodge, 2019).

International and regional treatment guidelines both play key roles in sharing evidence and pooling expertise. Regional guidelines developed for specific countries can be tailored to cultural considerations and the healthcare system of the country, while international guidelines can prevent the duplication of efforts and aid the development of regional guidelines. Rigorously prepared treatment guidelines are particularly important in rare diseases, as clinicians are often unfamiliar with the conditions and are therefore reliant on guidelines to direct treatment decisions (Pai et al., 2015). However, rare disease guidelines are frequently scarce due to lack of evidence in the literature as well as the resource‐intensive nature of guideline development.

To prepare a high‐quality guideline, robust evidence must be generated, such as through rigorous expert consensus and systematic literature review (SLR) (Guyatt et al., 2008; Pai et al., 2015). Increased incentives to encourage guideline development in the Nordics have recently been highlighted as part of the Innovative Nordic Health and Welfare Solutions programme, which has called for further collaboration in the development of treatment guidelines in the Nordic region (Denmark, Finland, Iceland, Norway, and Sweden) (Nordic Innovation, 2015). These countries lie in close geographical proximity to each other and are reasonably similar in terms of their economies, population density, healthcare and social systems, living standards, and cultures (Akehurst et al., 2017; Brouwers et al., 2016). Their similarities may facilitate increased collaboration, specifically in the field of rare diseases, which could make it possible to create holistic treatment guidelines while enabling resource pooling (Det Nationale Institut for Kommuners og Regioners Analyse og Forskning, 2015).

In light of these considerations, the objective of this targeted literature review (TLR) was to perform a descriptive analysis of country‐specific and international treatment guidelines regarding the pharmacological management of seizures in DS, LGS, RTT, TSC, and CDD in the Nordics. More specifically, the research aimed to:

- Determine the availability of country‐specific and international treatment guidelines for all five epileptic disorders
- Describe the methodology used to develop individual existing guidelines
- Assess the extent of collaboration between authors of the identified guidelines; and
- Report the frequency and patterns of existing treatment recommendations for DS, LGS, RTT, TSC, and CDD.

## 2 METHODS

### 2.1 Search strategy

A TLR was performed between November 5 and December 4, 2020. Online information sources were manually searched in accordance with prespecified search criteria to identify relevant treatment guidelines. The search strategies used for each information source and the dates of searches are summarized in Table S1. The search strategy included searches of the following sources: Google, Guideline Central, Orphanet, National Organisation for Rare Disorders (NORD), and International League Against Epilepsy (ILAE). Websites of national medicines agencies and HTA bodies for the following countries were also searched: Denmark, Finland, Iceland, Norway, and Sweden. Each database was queried with search terms appropriate for its search functionality (e.g., Boolean operators were used where possible) and the specificity of the database; searches were filtered for guidelines where possible. Search terms included combinations of free‐text and terms for each indication of interest, which were translated into the relevant language where applicable. When considering guidelines on the use of cannabidiol, no distinction was made between regulated and nonregulated formulations of cannabidiol in the search strategy.

### 2.2 Review process

Each record identified through the searches was screened for eligibility according to criteria defined using a PICOS (Population, Intervention, Comparators, Outcomes, and Study design) approach, as presented in Table S2. Briefly, eligible publications were guidelines or guidance reporting routine pharmacological management of seizures in patients with DS, LGS, TSC, RTT, or CDD in the countries of interest described previously. Eligible publications were classified as "International" if they were developed for multiple countries or did not specify to which countries they pertained. In addition to guidelines produced by HTA bodies, the review captured technology appraisal guidance following technology assessments.

Search results were screened by a single reviewer. Where the applicability of the inclusion criteria was unclear, the record was assessed by a second reviewer. Where possible, reviewers who were fluent or had a high level of proficiency in a relevant language were responsible for the identification, screening, and extraction of any guideline documents not published in the English language. For languages in which reviewers were not proficient, the online translation software DeepL^®^ (https://www.deepl.com/en/translator) was used.

### 2.3 Data extraction and analyses

Guidelines presenting relevant data were extracted into a predefined extraction grid. Information extracted for each guideline included: publication date and planned revision date; organization that developed the guideline; author names and affiliations; methodology used for the development of guidelines, including use of literature reviews and expert consultation; population(s) addressed; pharmacological recommendations by treatment stage and seizure subtype and references to other guidelines, HTA assessments/regulatory body decisions, and compiled literature sources (including SLRs, meta‐analyses, and electronic databases).

Descriptive analyses were performed in Microsoft Excel^®^ to examine the distribution of identified guidelines across the countries of interest, the methodologies used to develop the treatment guidelines, and the cross‐referencing of treatment recommendations made within other guidelines. The authors involved in developing each of the guidelines identified in this study (including guidelines for multiple indications) were mapped into a network, using R version 3.5.1 to visualize whether authors were contributing to > 1 guideline and if so, to measure the extent of collaboration between these authors, both on a national and international level.

To assess the patterns of positive and negative pharmacological treatment recommendations for each indication, further descriptive analyses were performed. A positive recommendation was defined as an individual ASM that was recommended for use in a specific indication, irrespective of the line of treatment (e.g., first line) or whether the treatment was adjunctive; a negative recommendation was defined as an individual ASM treatment that was highlighted as a potential option by a guideline but not recommended for use (for any reason) in a specific indication, irrespective of the line of treatment or whether the treatment was adjunctive.

## 3 RESULTS

### 3.1 Characteristics of included guidelines

**FIGURE 1:**
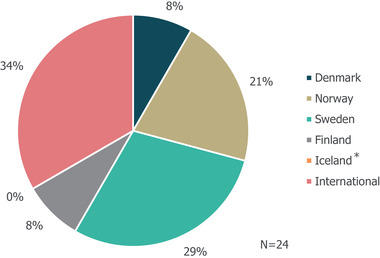
Geographies of identified guidelines. *No guidelines were identified for use in Iceland. The geography of guideline use refers to the country for which that the guidance was specifically developed

A total of 24 eligible records were included in the review following removal of duplicate results (Figure S1). More detailed information regarding each of the guidelines is presented in Table S3. Most guidelines were country specific; however, eight guidelines were classified as "International" (Figure 1). The countries with the highest number of identified guidelines were Sweden (7; 29%) and Norway (5; 21%). No national guidelines were identified for use in Iceland, and two of the international guidelines were specifically developed for Europe. Swedish guidelines appeared to be most likely to be specific to a particular indication with 4/7 focusing on one of the five investigated indications, while Danish and Finnish guidelines provided guidance that gave recommendations for more than one indication.

### 3.2 Evidence base and methodology for guideline development

**FIGURE 2:**
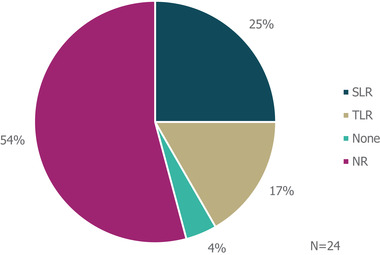
Types of literature review performed to inform guideline development “None” refers to guidelines in which a literature review was explicitly not used. *Abbreviations*: NR, not reported; SLR, systematic literature review; TLR, targeted literature review

**FIGURE 3:**
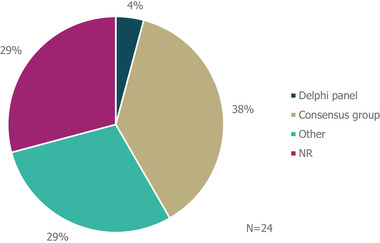
Types of expert consultation performed to inform guideline development. “Other” refers to working groups or targeted expert interviews. *Abbreviation*: NR, not reported

The majority of guidelines identified (54% [13/24]) did not specify whether literature reviews were used to inform guideline development (Figure 2); another guideline (4%) explicitly stated that a literature review was not used as part of the development process. The remaining guidance documents involved either systematic (25% [6/24]) or targeted (17% [4/24]) literature searches. Details on expert consultation were not reported by 7/24 guidelines (29%; Figure 3); one guideline (4%) involved a Delphi panel to inform guidance, while nine guidelines (38%) were based on formal consensus group exercises; the remaining seven guidelines (29%) utilized other forms of expert consultation, such as working groups or targeted expert interviews. Although 9/24 (38%) of guidelines reported the use of a combined development approach consisting of a literature review and expert consultation, only one explicitly used an SLR and Delphi panel in combination.

**FIGURE 4:**
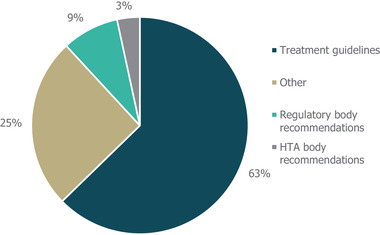
Guideline cross‐referencing to other treatment guidelines and regulatory/HTA recommendations. Cross‐referencing refers to the number of different treatment guidelines, regulatory body recommendations, HTA body recommendations or other references that were cited within the guidelines identified in this study, either in the body of the guideline text or in accompanying reference lists. “Other” references included Cochrane systematic literature reviews, a consensus conference report, one randomized controlled trial and one textbook. *Abbreviation*: HTA, health technology assessment

An analysis of citations of other guidelines, HTA and regulatory decisions, and other pooled literature sources including SLRs revealed that citations were mainly of existing treatment guidelines (63%) or “other” sources (25%), with the majority of these being SLRs included in the Cochrane Database of Systematic Reviews (Figure 4). Similarly, the two documents with the most individual references (six and three, respectively) were a publication of pediatric epilepsy treatment guidelines based on expert consensus opinion (Wheless et al., 2007) and a Cochrane SLR on the treatment of LGS (Hancock, 2003). Two guidelines each referenced an HTA body recommendation, and recommendations by regulatory bodies were referenced five times, by a total of three guidelines.

### 3.3 Extent of author collaboration

**FIGURE 5:**
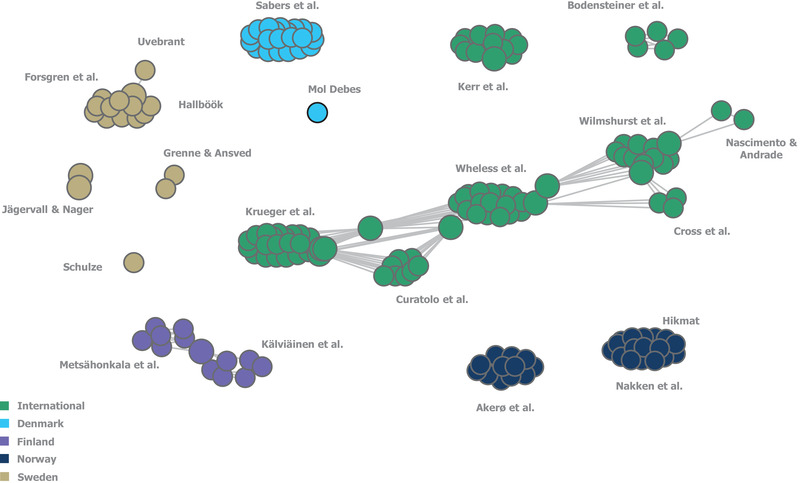
Map of collaboration between the author groups of included guidelines. Each individual circle represents one author of a guideline. Each “cluster” represents the group of authors that developed one guideline. Each cluster is labeled with the names of its respective first author(s). Guidelines which share one or more authors between them are connected by grey lines, with single circles between guideline clusters representing the individuals who authored both guidelines in question. Guidelines were classified as "International" if they were developed either for multiple countries or did not specify to which countries they pertained. Guidelines for which author names were not reported have not been included in this figure

To identify potential networks of clinical experts, named authors of published guidance documents and their connections through co‐authorship were visualized through an author map (Figure 5). Authors mostly worked within contained national groups, displaying only occasional connections between author groups, with most connections due to a single author being involved in two different guidelines. The author groups preparing international guidelines showed a higher degree of interconnectivity.

### 3.4 Treatment recommendations for DS

**FIGURE 6:**
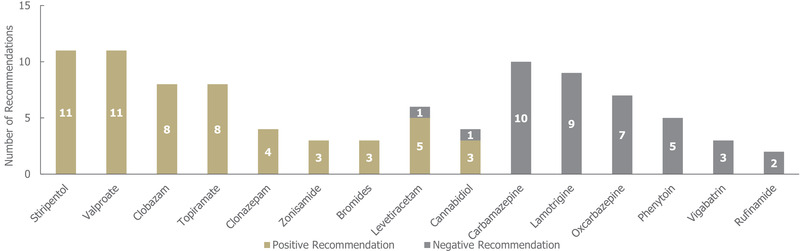
Treatment recommendations for Dravet syndrome, *n* = 99 (61 positive and 38 negative recommendations) from 12 guidelines. Positive recommendation: use of an individual ASM that was recommended for use in a specific indication, irrespective of the line of treatment (e.g., first line) or whether the treatment was adjunctive; Negative recommendation: an individual ASM treatment that was highlighted as a potential option by a guideline but not recommended by a guideline for use (for any reason) in a specific indication, irrespective of the line of treatment or whether the treatment was adjunctive. *Abbreviation*: ASM, anti‐seizure medication

In the 12 guidelines identified for DS, a total of 99 treatment recommendations were made (across 19 individual treatments, irrespective of the line of treatment; Figure 6). Of these treatment recommendations, a higher proportion were positive (62%; 61/99) than negative (38%; 38/99).

Out of the 19 treatments included in recommendations, 11 received exclusively positive recommendations for use in DS, of which stiripentol, valproate, clobazam, and topiramate had the highest number (≥8 each). Of these, only stiripentol has been approved by the Food and Drug Administration (FDA) and European Medicines Agency (EMA) for the treatment of seizures in DS (Food & Drug Administration, 2018). Several treatments (6/19) received exclusively negative recommendations for use in DS, of which carbamazepine, lamotrigine, and oxcarbazepine had the highest number (≥7 each). Of the 61 total positive treatment recommendations for DS, 43 (70%) were recommended for a specific line of treatment (32 for first line, 11 for second line; see Table S4.) Sodium valproate received the highest number of positive first‐line recommendations (10), followed by clobazam and stiripentol (six each; stiripentol is approved only as an add‐on therapy to sodium valproate and clobazam) (National Institute for Health & Care Excellence, 2017). Stiripentol received the highest number of positive second‐line recommendations (three). There were only two seizure type‐specific recommendations for DS, which were positive recommendations for the use of levetiracetam and topiramate in focal seizures, and no negative seizure type‐specific recommendations.

### 3.5 Treatment recommendations for LGS

**FIGURE 7:**
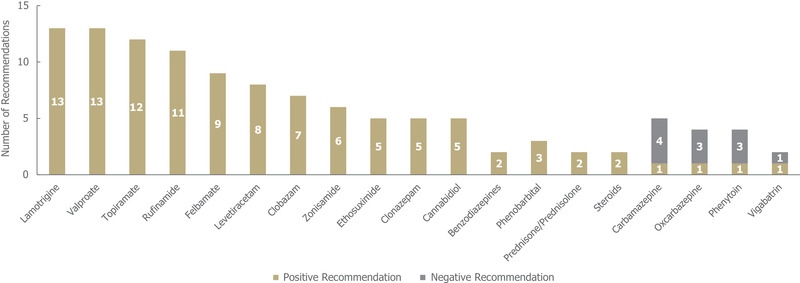
Treatment recommendations for Lennox‐Gastaut syndrome, *n* = 126 (114 positive and 11 negative treatment recommendations) from 14 guidelines. Positive recommendation: use of an individual ASM treatment that was recommended for use in a specific indication, irrespective of the line of treatment (e.g., first line) or whether the treatment was adjunctive; negative recommendation: an individual ASM treatment that was highlighted as a potential option by a guideline but not recommended by a guideline for use (for any reason) in a specific indication, irrespective of the line of treatment, or whether the treatment was adjunctive. *Abbreviations*: ACTH, adrenocorticotropic hormone; ASM, anti‐seizure medication

In the 14 guidelines identified for LGS, a total of 126 individual treatment recommendations were made irrespective of line of treatment (Figure 7). Of these 126 individual recommendations, 114 (90%) were positive and 11 (10%) were negative. Most of the medications that were included in recommendations (85% [22/26]) received exclusively positive recommendations for LGS. Of these 22 medications, lamotrigine, valproate, topiramate, and rufinamide received the highest number of positive recommendations (with ≥11 each, and no negative recommendations; Figure 7). Within this group, topiramate and rufinamide have been specifically approved for the treatment of epilepsy in LGS, in addition to felbamate, clobazam, and cannabidiol. Carbamazepine received the highest number of individual negative recommendations (four) across the guidelines.

Out of the 114 positive treatment recommendations, 44 (39%) were recommended for a specific treatment line for LGS (Table S5). Sodium valproate received the highest number of positive recommendations as a first‐line therapy (11), whereas lamotrigine received the highest number of positive recommendations as a second‐line therapy (four). Additionally, there were 25 seizure type‐specific recommendations for LGS, which covered a wide range of seizure types, including absence (one), atonic (four), atypical absence (13), crisis episode (three), generalized (three) and tonic‐clonic (one). The most frequent seizure type‐specific recommendations (each receiving two) were positive recommendations for ethosuximide, levetiracetam, phenobarbital, and zonisamide in atypical absence seizures.

### 3.6 Treatment recommendations for TSC

**FIGURE 8:**
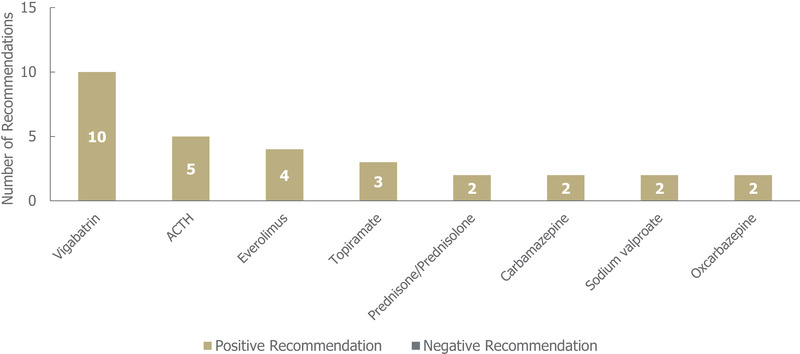
Treatment recommendations for tuberous sclerosis complex, *n* = 33 (32 positive and 1 negative treatment recommendations) from nine guidelines. Positive recommendation: use of an individual ASM treatment that was recommended for use in a specific indication, irrespective of the line of treatment (e.g., first line) or whether the treatment was adjunctive; negative recommendation: an individual ASM treatment that was highlighted as a potential option by a guideline but not recommended by a guideline for use (for any reason) in a specific indication, irrespective of the line of treatment, or whether the treatment was adjunctive. *Abbreviations*: ACTH, adrenocorticotropic hormone; ASM, anti‐seizure medication

In the nine guidelines identified for TSC, a total of 33 individual treatment recommendations were made for 11 treatments (irrespective of the line of treatment; Figure 8). Of these, 32/33 (97%) were positive. Out of the 11 treatments for which recommendations were made, 10 received exclusively positive recommendations for use in TSC, of which vigabatrin, adrenocorticotropic hormone (ACTH) and everolimus had the highest number (≥4 each). Cannabidiol received one negative recommendation for use in TSC.

Out of the 32 total positive treatment recommendations for TSC, 23 (72%) were recommended for a specific line of treatment (10 for first line, 13 for second line; see Table S6. Vigabatrin received the highest number of positive first‐line recommendations (eight), followed by oxcarbazepine and ACTH (one each). ACTH received the highest number of positive second‐line recommendations (four). There were multiple seizure type‐specific recommendations for TSC, which were positive recommendations for infantile spasms (16), focal seizures (five), and treatment‐refractory seizures (one).

### 3.7 Treatment recommendations for RTT and CDKL5 deficiency disorder

No treatment guidelines for routine pharmacological seizure management in RTT or CDD were identified.

## 4 DISCUSSION

This review provides a comprehensive overview of country‐specific treatment guidelines and recommendations for managing seizures in DS, LGS, RTT, TSC, and CDD in the five countries of the Nordics region (Denmark, Finland, Iceland, Norway, and Sweden). In summary, the review identified a limited use of “gold standard” methodologies used in guideline development (i.e., using a combination of an SLR and Delphi panel); limited collaboration between national author groups; a range of treatment recommendations for DS, LGS, and TSC and no treatment recommendations for CDD and RTT. Most guidelines were country‐specific, while a third were classified as “international.” Swedish guidelines appeared to be most likely to specify a particular indication, while Danish and Finnish guidelines featured more than one indication (DS and LGS were often grouped with 8/12 guidelines that reported on treatment recommendations for DS also including recommendations for LGS). No guidelines were identified which were specifically prepared for Iceland, whose relatively small population may heighten the challenge of developing national treatment guidelines for these rare disorders. Indeed, unlike the other four countries in this review, Iceland does not have a national chapter of the ILAE, which has produced international treatment guideline recommendations. This suggests that further collaboration on treatment guidelines within the Nordics region could be particularly beneficial for patients and clinicians in Iceland.

When considering the evidence base identified by the review, less than half of included guidelines described the development process in detail; 29% did not report on the methodology used to develop the guidance at all. Overall, there was a lack of reporting on whether literature reviews had been performed; however, reporting of methodology regarding expert opinion elicitations was more common (25% of guidance documents reported only on the type of expert consultation used, with no details regarding literature review methodology). The use of literature reviews in combination with expert consultation, where reported, varied considerably across guidelines and only one of the included guidelines used both an SLR and Delphi panel, which could be considered the “gold standard” for guideline development approaches (Khodyakov et al., 2017; World Health Organisation, 2014). These findings suggest that, while increased incentives are available to promote creation of guidelines in the Nordics, standardization and a shift towards more rigorous methods are needed in the development and reporting of treatment guidelines. Moreover, as indirect treatment comparisons of ASMs become increasingly available in published literature, it will be important to rigorously and transparently incorporate these results into the development of guidelines and recommendations (Strzelczyk & Schubert‐Bast, 2020). This will be especially important in the context of rare diseases, for which direct comparisons are much more challenging to obtain (Pavan et al., 2017).

Of the individual recommendations made by national guidelines, 78/182 (43%) were not accompanied by justification for the recommendation. In contrast, only 13/76 (17%) of the recommendations made by international guidelines were left unjustified. This may be linked to the fact that unlike the international guidelines, none of the country‐specific guidelines were published in peer‐reviewed journals. This further highlights the need for standardization in reporting of guidelines, even when those guidelines are not meant to undergo peer‐review and publication. Tools to facilitate the improvement of guideline reporting do already exist, for example, the AGREE checklist (Brouwers et al., 2016); these may therefore provide the basis for aligning guideline development practice on an international level and across (rare) disease indications.

This review identified links between author groups publishing international guidelines (Curatolo et al., Krueger et al., Wheless et al., Wilmshurst et al., Cross et al., and Nascimento and Andrade), while authors of national guidelines predominantly worked in contained groups, with only occasional connections between author groups. This suggests only modest collaboration between guideline authors in the Nordic region and that additional communication between national expert groups and supranational bodies could help to address the current lack of international guidelines for these disorders, particularly for RTT and CDD. Despite limited collaboration, however, treatment recommendation trends were similar across guidelines from different countries, which could partially be attributed to the widespread use of pivotal references such as the expert consensus developed by Wheless et al. (2007) or the Cochrane Database SLR by Hancock et al. (2013).

Treatment recommendations across the different indications were characterized by a high degree of heterogeneity regarding both the availability of guidance and specific recommendations within the individual indications. Conditions covered by a higher number of guidelines (i.e., LGS and DS) tended to have more conflicting recommendations, potentially reflecting the greater and more nuanced evidence base for these indications. In this regard, it should also be noted that wording around "limited evidence/experience" could be found across both positive and negative recommendations, indicating some variability in the way clinical evidence is evaluated and deemed sufficient for recommendations. While medications treating DS received the highest number of negative recommendations, likely due to multiple commonly used ASMs being clearly contraindicated, treatment recommendations for LGS were more conflicting, even though there were fewer negative treatment recommendations overall.

Indeed, overall treatment recommendations for DS showed a high level of consensus, with most treatments receiving either exclusively positive or negative recommendations. Cannabidiol was one of only two ASMs to receive mixed recommendations for the treatment of DS; in addition to three positive recommendations, it was negatively recommended in one guidance document due to the lack of a regulated preparation at the time of the guideline's publication (UpToDate, 2017), while the low quality of evidence regarding levetiracetam's efficacy resulted in one negative recommendation (it was also positively recommended five times) (Metsähonkala et al., 2020; Mol Debes Nea, 2014; Nascimento & Andrade, 2017; Nakken Kea, 2016; Helsebiblioteket, 2016; Hikmat, 2015). Valproate, first licensed in 1967, continued to be recommended positively across all guidelines for DS treatment despite the risk of teratogenic effects (European Medicines Agency, 2018a, 2018b).

The highest number of treatment recommendations were identified for LGS, and no medication has been unanimously negatively recommended for the treatment of LGS, although carbamazepine, oxcarbazepine, phenytoin, and vigabatrin all received negative recommendations due to their potential to aggravate seizures. The high number of treatment recommendations may reflect the fact that LGS can be challenging to treat due to patients experiencing multiple different, ASM‐resistant seizure types.

A comparatively low number of treatment recommendations for TSC was identified. However, while treatment guidelines for DS and LGS often reported on both conditions, 9/10 guidelines for TSC exclusively looked at this condition. As multiple organs are affected by TSC, guidelines focusing on TSC may reflect the complex management required, which includes seizure management (National Organization for Rare Disorders, 2019). Seizures can vary significantly for each patient, and many patients develop treatment‐resistant seizures, making individualized ASM combinations more likely (National Organization for Rare Disorders, 2019).

This review highlights a lack of both international and Nordic‐specific treatment guidelines for RTT and CDD, despite an increased drive from Nordic countries to improve collaboration in treatment guideline development. This may be linked to the difficulty of developing treatment guidelines for rare diseases in general, both due to low disease prevalence and lack of resources. While increased collaboration between countries can lead to significant cost‐savings over time when developing multiple guidelines in a disease area (Nordic Innovation, 2015), the lack of evidence in rare diseases may lead to these cost savings not being realized. There is therefore an urgent need for additional evidence in multiple rare epileptic conditions, in particular for both RTT and CDD.

Due to the time required for high‐quality data to be developed for newly authorized medications, there may be a delay between market authorization of a novel therapeutic and its broad inclusion in treatment recommendations. For example, fenfluramine received market authorization from the EMA for the treatment of DS in 2020 but was positively recommended in only one guidance document (European Medicines Agency, 2020). Cannabidiol has been included somewhat more rapidly into the treatment guideline literature, receiving five and three positive recommendations in LGS and DS, respectively, after obtaining market authorization for these disorders in 2019 (European Medicines Agency, 2019). In 2021, cannabidiol was further authorized for the treatment of TSC (European Medicines Agency, 2021), but has not been positively recommended for TSC treatment in any of the guidance documents included herein. These “gaps” between market authorization of new medications and their appearance in published treatment guidelines underscore the need for robust, timely guideline development, without which clinicians may struggle to stay abreast of the highly dynamic treatment landscape.

This study is subject to some limitations; the eligibility of all records was assessed by a single reviewer only, with a second adjudicating the decision when the applicability of the inclusion criteria was unclear. Due to the targeted nature of the review, searches were pragmatically focused on specialized databases and similar data sources. However, this did include a wide range of particularly relevant sources (both national and international) ranging from guideline repositories to HTA bodies, with supplementary searches via Google further minimizing the risk of missing relevant documents. While this well‐structured and comprehensive approach is likely to have resulted in the inclusion of the majority of relevant guidance for the indications of interest, a more systematic approach (including literature database searches and a dual review strategy for searches and data extraction) could have provided additional redundancy and further decreased the possibility of missing any relevant treatment recommendations. The time period of the review meant that only treatment guidelines published until December 2020 were captured here. Further to this, the identified treatment guidelines were published between May 2005 and October 2020 and any recommendations should therefore be interpreted in the context and date that they were made, as new research and drug approvals may necessitate updates to treatment guidelines. It should also be noted that details of the wider management of these disorders were not captured as part of the review, including emergency treatment of seizures, surgical interventions, or dietary modifications. Furthermore, in the analyses conducted within this review, therapies were not stratified by whether they were adjunctive. Further analysis could therefore be valuable to understand how recommendations vary based on whether a treatment is used adjunctively. For instance, for the treatment of both DS and LGS, cannabidiol has been evaluated as an add‐on therapy to clobazam use and was associated with a higher rate of seizure response in comparison to placebo when added to the existing treatment regimen of patients (Lattanzi et al., 2020).

The findings of this review emphasize the need for the development of further high‐quality guidance for the management of seizures with pharmacological therapy in DS, LGS, TSC, RTT, and CDD specific to the Nordic region. This should be influenced by an increased level of collaboration between countries, for example, by pooling of resources and expert knowledge, which may be particularly important for countries like Iceland where no country‐specific guidelines exist. In addition, the lack of treatment recommendations for RTT and CDD highlights an urgent need for further guidance on selection of an appropriate ASM regimen for these disorders to optimize seizure management.

## AUTHOR CONTRIBUTIONS

Substantial contributions to study conception and design: Kishan Vyas, Hannah Luedke, and Benjamin Ruban‐Fell. Substantial contributions to analysis and interpretation of the data: Kishan Vyas, Hannah Luedke, and Benjamin Ruban‐Fell. Drafting the article or revising it critically for important intellectual content: Kishan Vyas, Hannah Luedke, and Benjamin Ruban‐Fell. Final approval of the version of the article to be published: Kishan Vyas, Hannah Luedke, and Benjamin Ruban‐Fell.

## CONFLICT OF INTRESTS

Editorial and medical writing services were provided by Costello Medical. K. Vyas, employee of GW Pharma Ltd; H. Luedke, employee of Costello Medical; B. Ruban‐Fell, employee of Costello Medical.

### PEER REVIEW

The peer review history for this article is available at https://publons.com/publon/10.1002/brb3.2622

## Supporting information

Supporting information

## Data Availability

The data that support the findings of this study are available from the following resources available in the public domain:
Google (www.google.com) Guideline Central (https://www.guidelinecentral.com) Orphanet (https://www.orpha.net) National Organisation for Rare Disorders (NORD) (https://rarediseases.org) International League Against Epilepsy (ILAE) (https://www.ilae.org) Danish Medicines Agency [Denmark] (https://laegemiddelstyrelsen.dk/en/) Danish Health Authority (SST) [Denmark] (https://www.sst.dk/en/English) Amgros [Denmark] (https://www.amgros.dk/en/) Finnish Institute for Health and Welfare (THL) [Finland] (https://thl.fi/en/web/thlfi‐en) Finnish Medicines Agency (FIMEA) [Finland] (https://www.fimea.fi/web/en) Finnish Medical Society Duodecim [Finland] (https://www.duodecim.fi/english/) Icelandic Ministry of Health [Iceland] (https://www.stjornarradid.is/raduneyti/heilbrigdisraduneytid/) Icelandic Medicines Agency (IMA) [Iceland] (https://www.ima.is/) Icelandic Medicine Pricing and Reimbursement Committee (LGN) [Iceland] (https://verd.lyfjastofnun.is/) Norwegian Institute of Public Health (FHI) [Norway] (https://www.fhi.no/en/) Norwegian Medicines Agency [Norway] (https://legemiddelverket.no/english) Nye Metoder [Norway] (https://nyemetoder.no/) Swedish Medical Products Agency [Sweden] (https://www.lakemedelsverket.se/en) Dental and Pharmaceutical Benefits Agency (TLV) [Sweden] (https://www.tlv.se/) Swedish Agency for Health Technology Assessment and Assessment of Social Services (SBU) [Sweden] (https://www.sbu.se/en/) National Board of Health and Welfare (SOS) [Sweden] (https://www.socialstyrelsen.se/)
