# Supplementary material for: Nordic treatment guidelines for rare epileptic conditions: A literature review
Source: Brain Behav. 2022 Jun 28;12(7):e2622. doi: 10.1002/brb3.2622 (PMC9304844; doi:10.1002/brb3.2622)
Supplement: Supplementary file 1 — Supporting information [file BRB3-12-e2622-s001.docx]

SUPPLEMENTARY TABLES AND FIGURES

Figure S1. Literature review flowchart


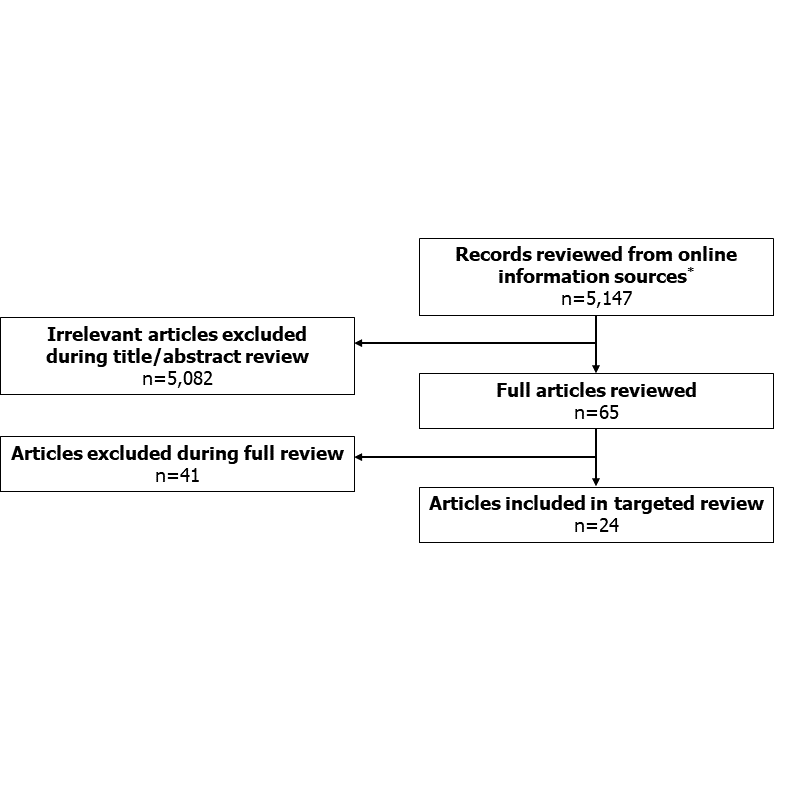


*Online information sources included: Guideline Central, National Organization for Rare Disorders (NORD), International League Against Epilepsy (ILAE), Orphanet**,** Google, Danish Medicines Agency, Danish Health Authority (SST), Amgros, Finnish Institute for Health and Welfare (THL), Finnish Medicines Agency (FIMEA), Finnish Medical Society Duodecim, Icelandic Ministry of Health, Icelandic Medicines Agency (IMA), Icelandic Medicine Pricing and Reimbursement Committee (LGN), Norwegian Institute of Public Health (FHI), Norwegian Medicines Agency, Nye Metoder, Swedish Medical Products Agency, Dental and Pharmaceutical Benefits Agency (TLV), Swedish Agency for Health Technology Assessment and Assessment of Social Services (SBU), National Board of Health and Welfare (SOS).

Table S1. Information sources and search strategies

| Information source | URL | Search date(s) | Search strategy† |
| --- | --- | --- | --- |
| Guideline Central | [https://www.guidelinecentral.com](https://www.guidelinecentral.com/) | 5^th^ November 2020 | The webpage was searched for the following terms (using the general search function):  *“dravet", “SMEI”, “severe myoclonus epilepsy of infancy”, “severe myoclonic epilepsy of infancy”, “lennox gastaut”, “tuberous sclerosis complex”, “bourneville”, “rett”, “cdkl5 deficiency”, “CDD”* |
| National Organisation for Rare Disorders (NORD) | [https://rarediseases.org](https://rarediseases.org/) | 5^th^ November 2020 | The webpage was searched for the following terms (using the advanced search function, searching ‘Rare Disease Database’ and ‘Physician Guides’ categories):  *"dravet", “SMEI”, “severe myoclonus epilepsy of infancy”, “severe myoclonic epilepsy of infancy”, “lennox gastaut”, “tuberous sclerosis complex”, “bourneville”, “rett”, “cdkl5 deficiency”,* *“CDD”* |
| International League Against Epilepsy (ILAE) | <https://www.ilae.org> | 5^th^ November 2020 | The webpage was searched for the following terms (using the general search function):  *"dravet", “SMEI”, “severe myoclonus epilepsy of infancy”, “severe myoclonic epilepsy of infancy”, “lennox gastaut”, “tuberous sclerosis complex”, “bourneville”, “rett”, “cdkl5 deficiency”*, *“CDD”* |
| Orphanet | [https://www.orpha.net](https://www.orpha.net/consor/cgi-bin/Disease_Search.php?lng=EN) | 5^th^ November 2020 | The webpage was searched for the following terms (using the “Disease name” setting):  *"dravet", “SMEI”, “severe myoclonus epilepsy of infancy”, “severe myoclonic epilepsy of infancy”, “lennox gastaut”, “tuberous sclerosis complex”, “bourneville”, “rett”, “cdkl5 deficiency”*, *“CDD”* |
| Google | [www.google.com](http://www.google.com) | 6^th–^18^th^ November 2020 | *“dravet", or “SMEI”, “severe myoclonus epilepsy of infancy”, or “severe myoclonic epilepsy of infancy”, or “lennox gastaut”, or “tuberous sclerosis complex”, or “bourneville”, or “rett”, or “cdkl5 deficiency”, or “CDD”* AND *“guideline”* AND *“Denmark”* or *“Finland”* or *“Iceland”* or *“Norway”* or *“Sweden”* |
|  |  | 18^th^–23^rd^ November 2020 | *“dravet", or “SMEI”, or “svær myoklon epilepsi hos børn”, or “lennox gastaut”, or “knoldsklerose kompleks”,* or *“bourneville”, or “rett”, or “cdkl5 mangelforstyrrelse”*, or*“CDD”* AND *“retningslinje”* AND *“Danmark”* |
|  |  | 27^th^ November 2020 | *“dravet", or “SMEI”, or "Imeväisiän vaikea myokloninen epilepsia”, or “lennox gastaut”, or “tuberoosiskleroosi”,* or *“bourneville”, or “rett”, or “cdkl5-häiriö”*, or*“CDD”* AND *“ohje”* AND *“Suomi”* |
|  |  | 27^th^ November 2020 | *"dravet", or “SMEI”, or "alvarleg vöðvakvilla flogaveiki”, or “lennox gastaut”, or “Hnjóskahersli”,* or *“bourneville”, or “rett”, or “cdkl5 deficiency”*, or*“CDD”* AND *“leiðbeiningar”* AND *“Ísland”* |
|  |  | 27^th^ November 2020 | *"dravet", or “SMEI”, or "Alvorlig myoklonisk epilepsi i spedbarnsalderen”, or “lennox gastaut”, or “tuberøs sklerosekompleks”,* or *“bourneville”, or “rett”, or “CDKL5-mangelforstyrrelse”*, or*“CDD”* AND *“retningslinje”* AND *“Norge”* |
|  |  | 27^th^ November 2020 | *"dravet", or “SMEI”, or "Svår myoklon epilepsy i spädbarn”, or “lennox gastaut”, or “tuberös skleroskomplex”,* or *“bourneville”, or “rett”, or “CDKL5-briststörning”*, or*“CDD”* AND *“riktlinje”* AND *“Sverige”* |
| National HTA Bodies/Medicine Agencies for Countries of Interest | | | |
| Danish Medicines Agency | <https://laegemiddelstyrelsen.dk/en/> | 1^st^ December 2020 | The webpage was searched for the following terms (using the general search function):  *"dravet", “SMEI”, “severe myoclonus epilepsy of infancy”, “severe myoclonic epilepsy of infancy”, “lennox gastaut”, “tuberous sclerosis complex”, “bourneville”, “rett”, “cdkl5 deficiency”*, *“CDD”, “svær myoklon epilepsi hos børn”, “knoldsklerose”* and *“cdkl5 mangelforstyrrelse”* |
| Danish Health Authority | <https://www.sst.dk> | 1^st^ December 2020 | The webpage was searched for the following terms (using the general search function):  *"dravet", “SMEI”, “severe myoclonus epilepsy of infancy”, “severe myoclonic epilepsy of infancy”, “lennox gastaut”, “tuberous sclerosis complex”, “bourneville”, “rett”, “cdkl5 deficiency”* ,*“CDD”, “svær myoklon epilepsi hos børn”, “knoldsklerose”* and *“cdkl5 mangelforstyrrelse”* |
| Amgros | <https://amgros.dk> | 1^st^ December 2020 | The webpage was searched for the following terms (using the general search function):  *"dravet", “SMEI”, “severe myoclonus epilepsy of infancy”, “severe myoclonic epilepsy of infancy”, “lennox gastaut”, “tuberous sclerosis complex”, “bourneville”, “rett”, “cdkl5 deficiency”* ,*“CDD”, “svær myoklon epilepsi hos børn”, “knoldsklerose”* and *“cdkl5 mangelforstyrrelse”* |
| Finnish Institute for Health and Welfare | <https://thl.fi/> | 1^st^ December 2020 | The webpage was searched for the following terms (using the general search function):  *"dravet", “SMEI”, “severe myoclonus epilepsy of infancy”, “severe myoclonic epilepsy of infancy”, “lennox gastaut”, “tuberous sclerosis complex”, “bourneville”, “rett”, “cdkl5 deficiency”*, *“CDD”, “Imeväisiän vaikea myokloninen epilepsia”, “tuberoosiskleroosi” and “CDKL5-häiriö”* |
| Finnish Medicines Agency | <https://www.fimea.fi> | 1^st^ December 2020 | The webpage was searched for the following terms (using the general search function):  *"dravet", “SMEI”, “severe myoclonus epilepsy of infancy”, “severe myoclonic epilepsy of infancy”, “lennox gastaut”, “tuberous sclerosis complex”, “bourneville”, “rett”, “cdkl5 deficiency”*, *“CDD”, “Imeväisiän vaikea myokloninen epilepsia”, “tuberoosiskleroosi” and “CDKL5-häiriö”* |
| Finnish Medical Society Duodecim | <https://www.kaypahoito.fi/> | 1^st^ December 2020 | The webpage was searched for the following terms (using the general search function):  *"dravet", “SMEI”, “severe myoclonus epilepsy of infancy”, “severe myoclonic epilepsy of infancy”, “lennox gastaut”, “tuberous sclerosis complex”, “bourneville”, “rett”, “cdkl5 deficiency”*, *“CDD”, “Imeväisiän vaikea myokloninen epilepsia”, “tuberoosiskleroosi” and “CDKL5-häiriö”* |
| Ministry of Health Iceland | <https://www.government.is/ministries/ministry-of-health/> | 3^rd^ December 2020 | The webpage was searched for the following terms (using the general search function):  *"dravet", “SMEI”, “severe myoclonus epilepsy of infancy”, “severe myoclonic epilepsy of infancy”, “lennox gastaut”, “tuberous sclerosis complex”, “bourneville”, “rett”, “cdkl5 deficiency”*, *“CDD”, “alvarleg vöðvakvilla flogaveiki”,* and *“Hnjóskahersli”* |
| Icelandic Medicines Agency | <https://www.ima.is/> | 3^rd^ December 2020 | The webpage was searched for the following terms (using the general search function):  *"dravet", “SMEI”, “severe myoclonus epilepsy of infancy”, “severe myoclonic epilepsy of infancy”, “lennox gastaut”, “tuberous sclerosis complex”, “bourneville”, “rett”, “cdkl5 deficiency”*, *“CDD”, “alvarleg vöðvakvilla flogaveiki”,* and *“Hnjóskahersli”* |
| Icelandic Medicine Pricing and Reimbursement Committee | <https://www.lgn.is/> | 3^rd^ December 2020 | The webpage was searched for the following terms (using the general search function):  *"dravet", “SMEI”, “severe myoclonus epilepsy of infancy”, “severe myoclonic epilepsy of infancy”, “lennox gastaut”, “tuberous sclerosis complex”, “bourneville”, “rett”, “cdkl5 deficiency”*, *“CDD”, “alvarleg vöðvakvilla flogaveiki”,* and *“Hnjóskahersli”* |
| Norwegian Institute of Public Health | <https://www.fhi.no/> | 3^rd^ December 2020 | The webpage was searched for the following terms (using the general search function):  *"dravet", “SMEI”, “severe myoclonus epilepsy of infancy”, “severe myoclonic epilepsy of infancy”, “lennox gastaut”, “tuberous sclerosis complex”, “bourneville”, “rett”, “cdkl5 deficiency”*, *“CDD”, “Alvorlig myoklonisk epilepsi i spedbarnsalderen”, “alvorlig myoklonus-epilepsi i tidlig barndom”, “tuberøs sklerosekompleks” and “CDKL5-mangelforstyrrelse”* |
| Norwegian Medicines Agency | <https://legemiddelverket.no/> | 3^rd^ December 2020 | The webpage was searched for the following terms (using the general search function):  *"dravet", “SMEI”, “severe myoclonus epilepsy of infancy”, “severe myoclonic epilepsy of infancy”, “lennox gastaut”, “tuberous sclerosis complex”, “bourneville”, “rett”, “cdkl5 deficiency”*, *“CDD”, “Alvorlig myoklonisk epilepsi i spedbarnsalderen”, “alvorlig myoklonus-epilepsi i tidlig barndom”, “tuberøs sklerosekompleks” and “CDKL5-mangelforstyrrelse”* |
| Nye Metoder | <https://nyemetoder.no/> | 3^rd^ December 2020 | The webpage was searched for the following terms (using the general search function):  *"dravet", “SMEI”, “severe myoclonus epilepsy of infancy”, “severe myoclonic epilepsy of infancy”, “lennox gastaut”, “tuberous sclerosis complex”, “bourneville”, “rett”, “cdkl5 deficiency”*, *“CDD”, “Alvorlig myoklonisk epilepsi i spedbarnsalderen”, “alvorlig myoklonus-epilepsi i tidlig barndom”, “tuberøs sklerosekompleks” and “CDKL5-mangelforstyrrelse”* |
| Swedish Medical Products Agency | <https://www.lakemedelsverket.se/> | 4^th^ December 2020 | The webpage was searched for the following terms (using the general search function):  *"dravet", “SMEI”, “severe myoclonus epilepsy of infancy”, “severe myoclonic epilepsy of infancy”, “lennox gastaut”, “tuberous sclerosis complex”, “bourneville”, “rett”, “cdkl5 deficiency”*, *“CDD”, “Svår myoklon epilepsy i spädbarn”, “tuberös skleroskomplex” and “CDKL5-briststörning”* |
| Dental and Pharmaceutical Benefits Agency | <https://www.tlv.se/> | 4^th^ December 2020 | The webpage was searched for the following terms (using the general search function):  *"dravet", “SMEI”, “severe myoclonus epilepsy of infancy”, “severe myoclonic epilepsy of infancy”, “lennox gastaut”, “tuberous sclerosis complex”, “bourneville”, “rett”, “cdkl5 deficiency”*, *“CDD”, “Svår myoklon epilepsy i spädbarn”, “tuberös skleroskomplex” and “CDKL5-briststörning”* |
| Swedish Agency for Health Technology Assessment and Assessment of Social Services | <https://www.sbu.se/> | 4^th^ December 2020 | The webpage was searched for the following terms (using the general search function):  *"dravet", “SMEI”, “severe myoclonus epilepsy of infancy”, “severe myoclonic epilepsy of infancy”, “lennox gastaut”, “tuberous sclerosis complex”, “bourneville”, “rett”, “cdkl5 deficiency”*, *“CDD”, “Svår myoklon epilepsy i spädbarn”, “tuberös skleroskomplex” and “CDKL5-briststörning”* |
| National Board of Health and Welfare | <https://www.socialstyrelsen.se/> | 4^th^ December 2020 | The webpage was searched for the following terms (using the general search function):  *"dravet", “SMEI”, “severe myoclonus epilepsy of infancy”, “severe myoclonic epilepsy of infancy”, “lennox gastaut”, “tuberous sclerosis complex”, “bourneville”, “rett”, “cdkl5 deficiency”*, *“CDD”, “Svår myoklon epilepsy i spädbarn”, “tuberös skleroskomplex” and “CDKL5-briststörning”* |

†All results were screened for relevance, with the exception of Google searches, where the first 3 pages of search results for each search were screened for relevance.

**Abbreviations**: CDD, CDKL5 deficiency disorder; HTA, health technology assessment; ILAE, International League Against Epilepsy; NORD, National Organisation for Rare Disorders; SMEI, severe myoclonic epilepsy of infancy.

Table S2. Eligibility criteria

| Modified PICOS domain | Inclusion criteria | Exclusion criteria |
| --- | --- | --- |
| Population | Patients with the following epileptic conditions:   - Dravet syndrome - Lennox-Gastaut syndrome - Tuberous sclerosis complex - Rett syndrome - CDKL5 deficiency disorder | Conditions other than those listed |
| Intervention | Any | None |
| Outcomes | The document must have discussed the management of the conditions of interest in terms of pharmacological treatment pathways for routine seizure control | - Documents that did not discuss the management in terms of pharmacological treatment pathways - Emergency medication and surgical guidelines - Medications for the treatment of tumours (and not seizures) in tuberous sclerosis complex |
| Publication type | Guidelines or guidance documents | Publications other than guidelines |
| Other considerations | Specifically produced for use in:   - Denmark - Finland - Iceland - Norway - Sweden   International guidelines (i.e. guidelines produced for multiple countries that include or potentially include the countries of interest, or guidelines that do not specify which countries they pertain to) | Produced specifically for use in countries that were not of interest |

**Abbreviations**: EU, European Union; PICOS, Population, Intervention, Comparators, Outcomes, Study design.

Table S3. List of included guidelines

| Title | Developing bodies | Region | Date (latest revision) | Indication(s) | Reference |
| --- | --- | --- | --- | --- | --- |
| Referenceprogram for epilepsi | Secretariat for Reference Programs of the Danish National Board of Health | Denmark | May 2005 | - LGS - TSC | Sabers et al., 2007^1^ |
| Neuropaediatri - en pixibog | Neurogroup of the Herlev and Gentofte Hospital | Denmark | March 2014 | - DS - LGS | Mol Debes et al., 2014^2^ |
| Tuberous Sclerosis Complex Surveillance and Management: Recommendations of the 2012 International Tuberous Sclerosis Complex Consensus Conference | NR | International | October 2013 | - TSC | Krueger et al., 2013^3^ |
| Summary of recommendations for the management of infantile seizures: Task Force Report for the ILAE Commission of Pediatrics | ILAE, Commission for Pediatrics | International | June 2015 | - DS - TSC | Wilmshurst et al., 2015^4^ |
| Consensus guidelines in the management of epilepsy in adults with an intellectual disability | International Society for the Scientific Study of Intellectual Disability (IASSID) | International | August 2009 | - LGS | Kerr et al., 2009^5^ |
| Dravet Syndrome: Management and prognosis | UpToDate | International | September 2017 (Based on literature review current until February 2018) | - DS | Nascimento and Andrade, 2017^6^ |
| Tuberous sclerosis complex: Management and prognosis | UpToDate | International | March 2018 | - TSC | Bodensteiner et al., 2018^7^ |
| Management of epilepsy associated with tuberous sclerosis complex: Updated clinical recommendations | 2018 Paediatric Neurology Society | International | May 2018 | - TSC | Curatolo et al., 2018^8^ |
| Expert Opinion on the Management of Lennox–Gastaut Syndrome: Treatment Algorithms and Practical Considerations | NR | International | September 2017 | - LGS | Cross et al., 2017^9^ |
| Treatment of pediatric epilepsy: European expert opinion, 2007 | A grant from the Shainberg Foundation supported publication of the results | International | December 2007 | - TSC - LGS | Wheless et al., 2007^10^ |
| Epilepsiat (aikuiset) | Duodecim of the Finnish Medical Association and the Finnish Neurological Association | Finland | February 2020 | - DS - LGS | Kälviäinen et al., 2020^11^ |
| Epilepsiat ja kuumekouristukset (lapset ja nuoret) | Duodecim of the Finnish Medical Association and the Finnish Neurological Association | Finland | February 2020 | - DS - LGS | Metsähonkala et al., 2020^12^ |
| Kunnskapsbasert retningslinje om epilepsi (Knowledge-based treatment guidelines on epilepsy) | Special Hospital for Epilepsy (SSE) | Norway | November 2016 | - LGS - DS | Nakken et al., 2016^13^ |
| Dravets syndrom | National Competence Service for Rare Diagnoses (NKSD) | Norway | February 2019 | - DS | NKSD, 2019^14^ |
| Epilepsi | Norwegian Paediatric Association | Norway | 2016 | - LGS - DS | Norwegian Paediatric Association, 2016^15^ |
| Retningslinjer for behandling av barne epilepsi | Haukeland University Hospital | Norway | 2015 | - LGS   DS | Hikmat, 2015^16^ |
| Tuberøs sklerose (TS) - anbefalt oppfølging og behandling | Norwegian National Centre for Rare Diagnoses | Norway | August 2017 | - TSC | Akerø et al., 2017^17^ |
| Läkemedelsbehandling av epilepsi | Swedish Medical Products Agency | Sweden | October 2019 | - LGS - DS | Forsgren, et al., 2019^18^ |
| Dravets syndrom | Information Centre for Rare Health Conditions (Agrenska) | Sweden | February 2019 | - DS | Hallböök, 2019^19^ |
| Epilepsi hos barn | MEDIBAS | Sweden | October 2020 | - LGS - DS | Jägervall and Nager 2020^20^ |
| Lennox-Gastaut syndrome | MEDIBAS | Sweden | November 2019 | - LGS | Jägervall and Nager 2019^21^ |
| Epileptiska anfall hos barn och ungdomar – ett hjälpmedel för bedömning, diagnostik och behandling | Västra Götaland Regional Council | Sweden | January 2020 | - LGS - TSC | Schulze 2020^22^ |
| Tuberös skleros | MEDIBAS | Sweden | February 2018 | - TSC | Grenne and Answed, 2018^23^ |
| Tuberös skleros | Information Centre for Rare Health Conditions (Agrenska) | Sweden | December 2015 | - TSC | Uvebrant 2015^24^ |

**Abbreviations**: DS, Dravet syndrome, IASSID, International Society for the Scientific of Intellectual Disability; LGS, Lennox-Gastaut syndrome; NKSD, National Competence Service for Rare Diagnoses; SSE, Special Hospital for Epilepsy; TS, Tuberøs sklerose; TSC, tuberous sclerosis complex.

Table S4. Treatment line-specific recommendations for DS

| **Treatment line-specific recommendations (positive)** | | |  |
| --- | --- | --- | --- |
| **First-line** | **32** | **Second-line** | **11** |
| Valproate | 10 | Stiripentol | 3 |
| Clobazam | 6 | Levetiracetam | 2 |
| Stiripentol | 6 | Topiramate | 2 |
| Topiramate | 3 | Bromides | 1 |
| Cannabidiol | 2 | Clobazam | 1 |
| Clonazepam | 2 | Clonazepam | 1 |
| Benzodiazepines | 1 | Phenobarbital | 1 |
| Levetiracetam | 1 |  |  |
| Steroids | 1 |  |  |
|  |  |  |  |
| **Treatment line-specific recommendations (negative)** | | |  |
| **First-line** | **0** | **Second-line** | **0** |
| N/A |  | N/A |  |

**Abbreviations**: DS, Dravet syndrome; N/A, not applicable.

Table S5. Treatment line-specific recommendations for LGS

| **Treatment line-specific recommendations (positive)** | | |  |
| --- | --- | --- | --- |
| **First-line** | **25** | **Second-line** | **19** |
| Valproate | 11 | Lamotrigine | 4 |
| Lamotrigine | 5 | Rufinamide | 2 |
| Topiramate | 4 | Topiramate | 2 |
| Rufinamide | 3 | Clobazam | 2 |
| Benzodiazepines | 0 | Levetiracetam | 2 |
| Clobazam | 1 | Clonazepam | 2 |
| Levetiracetam | 1 | Zonisamide | 1 |
|  |  | Ethosuximide | 1 |
|  |  | Felbamate | 1 |
|  |  | Valproate | 1 |
|  |  |  |  |
| **Treatment line-specific recommendations (negative)** | | |  |
| **First-line** | **0** | **Second-line** | **0** |
| N/A |  | N/A |  |

**Abbreviations**: LGS, Lennox-Gastaut syndrome; N/A, not applicable.

Table S6. Treatment line-specific recommendations for TSC

| **Treatment line-specific recommendations (positive)** | | |  |
| --- | --- | --- | --- |
| **First-line** | **10** | **Second-line** | **13** |
| Vigabatrin | 8 | ACTH | 4 |
| Oxcarbazepine | 1 | Topiramate | 2 |
| ACTH | 1 | Prednisone/Prednisolone | 2 |
|  |  | Carbamazepine | 1 |
|  |  | Corticosteroids | 1 |
|  |  | Oxcarbazepine | 1 |
|  |  | Everolimus | 1 |
|  |  | Sodium valproate | 1 |
|  |  |  |  |
| **Treatment line-specific recommendations (negative)** | | |  |
| **First-line** | **0** | **Second-line** | **0** |
| N/A |  | N/A |  |

**Abbreviations**: ACTH, adrenocorticotropic hormone; TSC, tuberous sclerosis complex; N/A, not applicable.

**Supplementary References**

1. Sabers A, Rogvi-Hansen, B., Alving, J., Brøndum-Nielsen, K., Christensen, J., Dam, A.M., Erdal, J. et al. Sundhedsstyrelsen - Referenceprogram for epilepsi. 2005.

2. Mol Debes N on behalf of the Neurogroup of the Herlev and Gentofte Hospital. Neuropaediatri - en pixibog. 2014.

3. Krueger DA, Northrup H, Northrup H, et al. Tuberous Sclerosis Complex Surveillance and Management: Recommendations of the 2012 International Tuberous Sclerosis Complex Consensus Conference. *Pediatric Neurology*. 49(4):255-265.

4. Wilmshurst JM GW, Vinayan KP, Tsuchida TN, Plouin P, Van Bogaert P, Carrizosa J, Elia M, Craiu D, Jovic NJ, Nordli DR, Hirtz D, Wong V, Glauser T, Mizrahi EM, Cross JH. Summary of recommendations for the management of infantile seizures: Task Force Report for the ILAE Commission of Pediatrics. *Epilepsia*. 56(8):1185-1197.

5. Kerr M SM, Arvio M, Beavis J, Brandt C, Brown S, Huber B, Livanainen M, Louisse AC, Martin P, Marson AG, Prasher V, Singh BK, Veendrick M. Consensus guidelines in the management of epilepsy in adults with an intellectual disability. *Journal of Intellectual Disability Research*. 53(8):687-694.

6. Andrade DM NF, Nordli DR. Dravet Syndrome: Management and prognosis. <https://medsmart.shop/UpToDate/d/topic.htm?path=dravet-syndrome-management-and-prognosis#H3649996041> Accessed: 04 December 2020

7. Randle S. Tuberous sclerosis complex: Management and prognosis. <https://www.uptodate.com/contents/tuberous-sclerosis-complex-management-and-prognosis> Accessed: 04 December 2020

8. Curatolo P, Nabbout R, Lagae L, et al. Management of epilepsy associated with tuberous sclerosis complex: Updated clinical recommendations. *European Journal of Paediatric Neurology*. 22(5):738-748.

9. Cross HJ, Auvin S, Falip M, Striano P, Arzimanoglou A. Expert Opinion on the Management of Lennox–Gastaut Syndrome: Treatment Algorithms and Practical Considerations. Review. *Frontiers in Neurology*. 8(505)

10. Wheless J CD, Arzimanoglou A, Carpenter D, Arzimanoglou A, Barbosa C, Berquin P, Campos-Castello J, Covanis A, de Saint Martin A, Des Portes V, Echenne B, Fazzi E, Fohlen M, Franzoni E, Gobbi G, Herranz JL, Holthausen H, Isnard H, Katsarou E, Kener Z, Korff C, Lagae L, Mancini J, Menache C, Mikati M, Misson J, Newton RW, N'Guyen S, Pedespan J, Pinard J, Papacostas S, Rating D, Quilis MR, Stephani U, Temudo T, Tuxhorn I, Van Bogaert P, Van Nieuwenhuizen O, Van Ryckevorsel K, Veggiotti P, Vigevano F, Zuberi S. . Treatment of pediatric epilepsy: European expert opinion, 2007. *Epileptic Disorders*. 9(4):353-412.

11. Kälviäinen R AH, Immonen A, Keränen T, Komulainen J, Komulainen S, Lamuoso S, Peltola J. Epilepsiat (aikuiset). Käypä hoito. <https://www.kaypahoito.fi/hoi50072#readmore> Accessed: 04 December 2020

12. Metsähonkala L GE, Gilbert O, Kirjavainen J, Komulainen J, Lähdesmäki T, Vieira P. Epilepsiat ja kuumekouristukset (lapset ja nuoret). Käypä hoito. <https://www.kaypahoito.fi/hoi50059#readmore> Accessed: 04 December 2020

13. Nakken KO KE, Aaberg KM, Taubøll E, Brodtkorb E, Engelsen B, Wilson J, Gerstner T, Hikmat O, Sandvig I, Henning S, Cammermeyer Haavardsholm K, Peersen H, Kleven G. Kunnskapsbasert retningslinje om epilepsi. <https://www.epilepsibehandling.no/index.php?action=showchapter&chapter=7S7KCFB3> Accessed: 04 December 2020

14. National Competence Service for Rare Diagnoses (NKSD). Dravets syndrom. <https://www.helsenorge.no/sykdom/sjeldne-diagnoser/sjeldne-epilepsidiagnoser/dravets-syndrom/#behandling> Accessed: 04 December 2020

15. Norwegian Paediatric Association. Epilepsi. <https://www.helsebiblioteket.no/pediatriveiledere?key=144642&menuitemkeylev1=5962&menuitemkeylev2=5973> Accessed: 04 December 2020

16. Hikmat O. Retningslinjer for behandling av barne epilepsi. <https://kvalitet.helse-bergen.no/docs/pub/dok29389.pdf> Accessed: 04 December 2020

17. Akerø A AJ, Bjerre A, et al. Tuberøs sklerose (TS) - anbefalt oppfølging og behandling. <https://oslo-universitetssykehus.no/seksjon/nasjonalt-kompetansesenter-for-sjeldne-epilepsirelaterte-diagnoser/Documents/Tuber%C3%B8s%20sklerose%20-%20veileder.pdf> Accessed: 04 December 2020

18. Forsgren L KE, Sundelin H, Söderberg Löfdal K, Källén K, Dahlin M, Zelano J, Hallböök T, Bjellvi J, Timby N, Tomson T, Strandberg M. . Läkemedelsbehandling av epilepsi <https://www.lakemedelsverket.se/48d84a/globalassets/dokument/behandling-och-forskrivning/behandlingsrekommendationer/bakgrundsdokument/bakgrundsdokumentation-epilepsi.pdf> Accessed: 04 December 2020

19. Hallböök T. Dravets syndrom. <https://www.socialstyrelsen.se/stod-i-arbetet/sallsynta-halsotillstand/dravets-syndrom/> Accessed: 04 December 2020

20. Jägervall M, Nager, A. Epilepsi hos barn. <https://medibas.se/handboken/kliniska-kapitel/pediatrik/tillstand-och-sjukdomar/neurologi/epilepsi-hos-barn/> Accessed: 04 December 2020

21. Jägervall M, Nager, A. Lennox-Gastaut syndrome. <https://medibas.se/handboken/kliniska-kapitel/pediatrik/tillstand-och-sjukdomar/neurologi/lennox-gastauts-syndrom/> Accessed: 04 December 2020

22. Schulze E. Epileptiska anfall hos barn och ungdomar – ett hjälpmedel för bedömning, diagnostik och behandling. <https://alfresco.vgregion.se/alfresco/service/vgr/storage/node/content/44574/Epileptiska%20anfall%20hos%20barn%20och%20ungdomar-ett%20hj%c3%a4lpmedel%20f%c3%b6r%20bed%c3%b6mning,%20diagnostik%20och%20behandling.pdf?a=false&guest=true> Accessed: 04 December 2020

23. Grenne B, Ansved, T. Tuberös skleros. <https://medibas.se/handboken/kliniska-kapitel/neurologi/tillstand-och-sjukdomar/arftliga-sjukdomar/tuberos-skleros/> Accessed: 04 December 2020

24. Uvebrant P. Tuberös skleros. <https://www.socialstyrelsen.se/stod-i-arbetet/sallsynta-halsotillstand/tuberos-skleros/> Accessed: 04 December 2020
